# Gestational Exposure to Endocrine-Disrupting Chemicals and Reciprocal Social, Repetitive, and Stereotypic Behaviors in 4- and 5-Year-Old Children: The HOME Study

**DOI:** 10.1289/ehp.1307261

**Published:** 2014-03-12

**Authors:** Joseph M. Braun, Amy E. Kalkbrenner, Allan C. Just, Kimberly Yolton, Antonia M. Calafat, Andreas Sjödin, Russ Hauser, Glenys M. Webster, Aimin Chen, Bruce P. Lanphear

**Affiliations:** 1 Department of Epidemiology, Brown University, Providence, Rhode Island, USA; 2 Zilber School of Public Health, University of Wisconsin-Milwaukee, Milwaukee, Wisconsin, USA; 3 Department of Environmental Health, Harvard School of Public Health, Boston, Massaschusetts, USA; 4 Cincinnati Children’s Hospital Medical Center, Department of Pediatrics, Cincinnati, Ohio, USA; 5 Centers for Disease Control and Prevention, National Center for Environmental Health, Division of Laboratory Sciences, Atlanta, Georgia, USA; 6 Faculty of Health and Sciences, Simon Fraser University, Burnaby, British Columbia, Canada; 7 Child and Family Research Institute, BC Children’s and Women’s Hospital, Vancouver, British Columbia, Canada; 8 Division of Epidemiology and Biostatistics, Department of Environmental Health, University of Cincinnati, Cincinnati, Ohio, USA

## Abstract

Background: Endocrine-disrupting chemicals (EDCs) may be involved in the etiology of autism spectrum disorders, but identifying relevant chemicals within mixtures of EDCs is difficult.

Objective: Our goal was to identify gestational EDC exposures associated with autistic behaviors.

Methods: We measured the concentrations of 8 phthalate metabolites, bisphenol A, 25 polychlorinated biphenyls (PCBs), 6 organochlorine pesticides, 8 brominated flame retardants, and 4 perfluoroalkyl substances in blood or urine samples from 175 pregnant women in the HOME (Health Outcomes and Measures of the Environment) Study (Cincinnati, OH). When children were 4 and 5 years old, mothers completed the Social Responsiveness Scale (SRS), a measure of autistic behaviors. We examined confounder-adjusted associations between 52 EDCs and SRS scores using a two-stage hierarchical analysis to account for repeated measures and confounding by correlated EDCs.

Results: Most of the EDCs were associated with negligible absolute differences in SRS scores (≤ 1.5). Each 2-SD increase in serum concentrations of polybrominated diphenyl ether-28 (PBDE-28) (β = 2.5; 95% CI: –0.6, 5.6) or *trans*-nonachlor (β = 4.1; 95% CI: 0.8–7.3) was associated with more autistic behaviors. In contrast, fewer autistic behaviors were observed among children born to women with detectable versus nondetectable concentrations of PCB-178 (β = –3.0; 95% CI: –6.3, 0.2), β-hexachlorocyclohexane (β = –3.3; 95% CI: –6.1, –0.5), or PBDE-85 (β = –3.2; 95% CI: –5.9, –0.5). Increasing perfluorooctanoate (PFOA) concentrations were also associated with fewer autistic behaviors (β = –2.0; 95% CI: –4.4, 0.4).

Conclusions: Some EDCs were associated with autistic behaviors in this cohort, but our modest sample size precludes us from dismissing chemicals with null associations. PFOA, β-hexachlorocyclohexane, PCB-178, PBDE-28, PBDE-85, and *trans*-nonachlor deserve additional scrutiny as factors that may be associated with childhood autistic behaviors.

Citation: Braun JM, Kalkbrenner AE, Just AC, Yolton K, Calafat AM, Sjödin A, Hauser R, Webster GM, Chen A, Lanphear BP. 2014. Gestational exposure to endocrine-disrupting chemicals and reciprocal social, repetitive, and stereotypic behaviors in 4- and 5-year-old children: the HOME Study. Environ Health Perspect 122:513–520; http://dx.doi.org/10.1289/ehp.1307261

## Introduction

Autism spectrum disorders (ASDs) affect approximately 1% of U.S. children and are characterized by impaired interpersonal behavior or communication and repetitive or stereotypic behaviors that begin before 3 years of age [American Psychiatric Association 2000; Centers for Disease Control and Prevention (CDC) 2012]. Two lines of evidence suggest that the endocrine system plays a role in the etiology of ASDs. First, males are four times as likely to be diagnosed with ASDs as females (CDC 2012). Second, adrenal, gonadal, and thyroid hormones play an important role in fetal neurodevelopment (Auyeung et al. 2010; Henrichs et al. 2013; Ronald et al. 2010).

*In utero* environmental exposures may increase the risk of ASDs (Landrigan et al. 2012). Specifically, it has been proposed that endocrine-disrupting chemicals (EDCs) may alter endogenous hormone axes to interfere with steroid-dependent neurodevelopment and modify the risk of ASDs (Braun 2012). Pregnant women are exposed to a mixture of EDCs during pregnancy, including some that may increase the risk of childhood behavior disorders (de Cock et al. 2012; Landrigan et al. 2012; Woodruff et al. 2011). Isolating the potential effect of one EDC exposure from another is difficult when exposures are correlated due to common sources. For instance, serum levels of individual polychlorinated biphenyl (PCB) congeners are correlated with one another as well as with some organochlorine (OC) pesticides (Meeker et al. 2009).

Despite the importance of identifying modifiable risk factors for ASDs and large number of EDCs in our environment, few epidemiological studies have examined the relationship between gestational EDC exposures and ASDs (Miodovnik et al. 2011; Roberts et al. 2007). Thus, epidemiological studies with multiple EDC exposure biomarkers employing robust statistical methods are needed to screen components of the chemical mixtures humans are exposed to and identify the effect of individual EDCs from correlated co-pollutants.

To address this important research need, we used a semi-Bayesian hierarchical regression model to estimate associations between prenatal blood or urine concentrations of 52 suspected EDCs and autistic behaviors at 4 and 5 years of age in a prospective birth cohort of 175 mothers and their children.

## Methods

*Study participants*. The Health Outcomes and Measures of the Environment (HOME) Study is a prospective birth cohort from the greater Cincinnati, Ohio, metropolitan area designed to study the relationship between low-level environmental chemical exposure and children’s growth and development. Extensive data including biological specimens, environmental samples, interviews, psychometric tests, and anthropometric measurements were collected during pregnancy and at annual clinic and home visits when the children were between 1 and 5 years of age. We recruited pregnant women from seven prenatal clinics associated with three hospitals in the Cincinnati area from March 2003 to January 2006. Eligibility criteria at enrollment included *a*) 16 ± 3 weeks of pregnancy, *b*) age ≥ 18 years, *c*) residence in a home built before 1978, *d*) no history of HIV infection, and *e*) no medications taken for seizure or thyroid disorders. All women provided written informed consent for themselves and their children after the study protocols had been explained. The institutional review boards (IRBs) of Cincinnati Children’s Hospital Medical Center and the cooperating delivery hospitals approved this study. The CDC IRB relied on the determinations made by the other IRBs.

*Environmental chemical biomarkers*. Women provided spot urine samples in polypropylene cups at their prenatal care clinic visits around 16 and 26 weeks of pregnancy. At these same visits and within 24 hr of delivery, a serum sample was obtained via venipuncture. All samples were refrigerated until they were processed and aliquoted, after which they were stored at –20^o^C until shipped on dry ice to the CDC for analysis.

We measured concentrations of 70 suspected EDCs in either maternal serum (*n* = 60) or urine (*n* = 10) using sensitive and specific isotope dilution liquid or gas chromatography mass spectrometry (see Supplemental Material, Table S1) (Jones et al. 2012; Kuklenyik et al. 2005; Silva et al. 2007; Ye et al. 2008). Concentrations below the limit of detection were assigned a value of the LOD/_√_^–^2 (Hornung and Reed 1990). Our analyses included 52 chemicals after excluding 16 that were infrequently detected (< 20% of samples with detectable values), PCB-180 (Pearson *r* > 0.95 with PCB-170), and mono(2-ethyl-5-oxohexyl) phthalate (MEOHP) [Pearson *r* > 0.95 with mono(2-ethyl-5-hydroxyhexyl) phthalate (MEHPP)] (see Supplemental Material, Table S1). The chemical with the higher median concentration was chosen when two chemicals were highly correlated.

The total (free plus conjugated) urinary concentrations of eight phthalate metabolites and bisphenol A (BPA) were creatinine-normalized in units of micrograms per gram creatinine to account for urine dilution, and log_10_-transformed before being averaged if a woman provided more than one sample. PCB, brominated flame retardant (BFR), perfluoroalkyl substance (PFAS), and OC pesticide concentrations were measured in the 16-week serum samples. A small number of women’s PFAS concentrations were measured in serum samples collected at gestational week 26 (*n* = 9) and birth (*n* = 3) because the volume of the 16-week serum sample was insufficient for analysis. Concentrations of PCBs, OC pesticides, and BFRs were lipid-normalized in units of nanograms per gram serum lipid.

Chemicals with detection frequencies ≥ 80% (Table 1) were log_10_-transformed to reduce the influence of outliers and treated as continuous variables. Chemicals with detection frequencies ≥ 20% and < 80% (*n* = 13) were analyzed as dichotomous variables (detectable vs. nondetectable concentrations). We then rescaled continuous concentrations so that the magnitude and precision of changes in Social Responsiveness Scale (SRS) score would be comparable for both continuous and dichotomous variables. Specifically, we divided continuous chemical concentrations by 2 times their SD (Gelman 2008) to generate rescaled variables with SD = 0.5, consistent with the SD of a binary variable with a probability of 0.5. Thus, the differences in SRS scores are presented for each 2-SD increase in exposure for continuous variables or as the difference between exposed and unexposed participants for dichotomous variables.

**Table 1:** Univariate statistics of urinary or serum endocrine disrupting chemicals concentrations during pregnancy among 175 Cincinnati, Ohio, women (2003–2006) and median concentrations U.S. women (NHANES 2003–2004).

Chemical^*a*^	Percent > LOD	GM (GSD)	5th percentile	25th percentile	Median	75th percentile	95th percentile	Median in NHANES women (2003–2004)
MBP	100	26.0 (1.9)	9.5	18	26	37	75	24
MiBP	99	5.1 (2.1)	1.5	3.0	5.6	8.6	17	4.0
MEP	100	143.3 (2.9)	25	70	133	286	1010	120
MBzP	98	10.3 (2.4)	3.2	5.8	11	17	48	10
MCPP	99	2.4 (1.8)	1.1	1.6	2.3	3.4	5.9	2.9
MEHP	89	5.2 (2.7)	< LOD	2.9	4.4	7.5	41	2.2
MEHHP	100	41.0 (2.4)	7.7	15	22	49	152	19
MECPP	100	27.6 (2.5)	13	21	35	70	191	31
BPA	96	2.1 (1.9)	0.8	1.4	2.0	3.1	6.6	2.7
PCB-28	82	0.8 (2.9)	< LOD	0.7	1.0	1.6	3.3	5.0
PCB-66	75	5.6 (2.7)	< LOD	< LOD	0.6	1.0	2.2	1.4
PCB-74	99	2.8 (1.8)	1.2	2.0	2.6	3.9	7.0	5.4
PCB-99	99	2.8 (1.8)	1.2	1.9	2.8	3.9	7.1	3.9
PCB-101	32	2.1 (2.9)	< LOD	< LOD	< LOD	0.5	1.5	1.6
PCB-105	93	1.1 (2.5)	< LOD	0.8	1.1	1.7	3.4	1.2
PCB-118	99	4.9 (2.0)	2.1	3.2	4.8	7.1	14	5.0
PCB-138/158	99	7.8 (2.0)	3.0	5.3	7.7	11	25	16
PCB-146	93	1.0 (2.6)	< LOD	0.8	1.1	1.7	4.1	2.3
PCB-153	100	11.1 (1.9)	4.3	7.6	11	15	35	22
PCB-156	96	1.6 (2.4)	0.5	1.0	1.6	2.5	6.1	3.4
PCB-157	51	3.3 (3.2)	< LOD	< LOD	0.4	0.7	1.6	0.9
PCB-167	59	3.9 (3.1)	< LOD	< LOD	0.5	0.7	1.8	0.9
PCB-170	100	2.8 (2.2)	0.9	1.8	2.8	4.2	9.4	6.3
PCB-172	34	2.2 (3.0)	< LOD	< LOD	< LOD	0.5	1.2	0.9
PCB-177	61	4.0 (3.1)	< LOD	< LOD	0.5	0.7	2.1	1.3
PCB-178	52	3.3 (3.2)	< LOD	< LOD	0.4	0.7	1.8	1.2
PCB-183	87	0.8 (3.0)	< LOD	0.6	1.0	1.4	2.8	1.7
PCB-187	98	2.1 (2.3)	0.7	1.5	2.1	3.3	7.1	4.6
PCB-194	92	1.2 (2.8)	< LOD	0.9	1.4	2.2	4.5	4.0
PCB-195	39	2.4 (3.1)	< LOD	< LOD	< LOD	0.6	1.1	0.6
PCB-196/203	96	1.6 (2.3)	0.5	1.1	1.6	2.5	4.8	3.3
PCB-199	93	1.2 (2.8)	< LOD	0.9	1.3	2.2	4.4	3.7
PCB-206	81	0.6 (3.2)	< LOD	0.6	0.8	1.2	2.4	2.3
PCB-209	35	2.2 (3.0)	< LOD	< LOD	< LOD	0.5	1.0	1.2
β-HCH	27	1.9 (2.8)	< LOD	< LOD	< LOD	1.9	4.4	< LOD
HCB	94	6.5 (1.9)	< LOD	5.5	7.0	9.0	13.8	16
*p’p’*-DDT	52	3.3 (3.2)	< LOD	< LOD	1.9	3.2	6.2	< LOD
*p’p’*-DDE	100	71.6 (1.8)	31	51	67	93	182	206
Oxychlordane	90	4.4 (2.5)	< LOD	3.5	5.1	7.2	13	11
*trans*‑Nonachlor	97	7.5 (2.1)	2.3	5.1	7.4	12	25	15
BB-153	85	0.9 (3.7)	< LOD	0.6	1.1	1.9	4.5	2.0
PBDE-28	81	0.8 (4.0)	< LOD	0.5	1.1	1.8	4.2	1.0
PBDE-47	100	20.1 (2.7)	4.9	9.7	19	35	103	19
PBDE-85	49	3.1 (3.2)	< LOD	< LOD	< LOD	1.0	3.5	< LOD
PBDE-99	100	4.7 (2.9)	1.0	2.2	4.4	8.0	33	< LOD
PBDE-100	99	3.8 (3.0)	0.9	2.0	3.4	7.9	25	3.2
PBDE-153	99	5.1 (3.1)	1.3	2.4	4.2	9.0	54	4.0
PBDE-154	42	2.6 (3.1)	< LOD	< LOD	< LOD	0.9	2.8	0.8
PFOA	100	5.6 (1.7)	2.5	3.8	5.5	7.6	13	3.6
PFOS	100	13.1 (1.6)	5.7	9.3	13	18	27	18
PFNA	100	0.9 (1.5)	0.5	0.7	0.9	1.2	1.9	0.9
PFHxS	100	1.5 (2.0)	0.5	0.9	1.6	2.4	5.0	1.6
Abbreviations: BB-153, 2,2’,4,4’,5,5’-hexabromobiphenyl; GM, geometric mean; GSD, geometric standard deviation; MBP, mono-*n*-butyl-phthalate; MBzP, monobenzyl phthalate; MCPP, mono(3-carboxypropyl) phthalate; MECPP, mono(2-ethyl-5-carboxypentyl) phthalate; MEHP, mono(2-ethylhexyl) phthalate; MEP, monoethyl phthalate; MiBP, monoisobutyl phthalate; PFHxS, perfluorohexane sulfonate; PFNA, perfluorononanoate; PFOA, perfluorooctanoate; PFOS, perfluorooctane sulfonate. ^***a***^Concentrations are displayed in units of ng/g lipds (PCBs, PBDEs, and OC pesticides), μg/g creatinine (phthalates and BPA), and μg/L (PFAS).								

*Autistic behaviors*. Mothers completed the SRS (Constantiono 2005) up to two times in our study clinic when their children were 4 and 5 years of age. The SRS is a valid, reliable, and sensitive measure of interpersonal behaviors, communication, and repetitive or stereotypic behaviors (Bolte et al. 2008; Constantino 2005). The SRS assesses autistic behaviors along a continuum, rather than an “all or none” diagnosis, using 65 Likert-scale questions that are summed and transformed into a total *T*-score (mean ± SD, 50 ± 10 in the normative sample). Higher scores indicate more autistic behaviors, with *T*-scores ≥ 60 considered indicative of clinically significant deficiencies in reciprocal social behavior, and *T*-scores ≥ 75 being consistent with a clinical diagnosis of ASDs. However, we examined continuous SRS scores because of the small number of children with scores ≥ 60 (*n* = 22).

*Confounding variables*. We adjusted for the following potential confounding variables that might be associated with both environmental chemical exposures and autistic behaviors based on biological plausibility and prior knowledge. Maternal demographic and perinatal factors, including maternal age at delivery, race, marital status, education, parity, insurance status, employment, household income, and prenatal vitamin use were obtained using structured interviews and chart reviews conducted by trained research staff. Depressive symptoms during the second trimester were measured with the Beck Depression Inventory-II (Beck et al. 1996). Maternal Full-Scale IQ was measured using the Vocabulary and Matrix Reasoning subtests of the Wechsler Abbreviated Scale of Intelligence (Wechsler 1999). Caregiving environment was assessed when children were 1 year old using the HOME (Home Observation for Measurement of the Environment), an in-home semistructured interview and observational tool that measures the quality and quantity of environmental stimulation and support (Caldwell and Bradley 2003). Serum cotinine, a tobacco smoke exposure biomarker, was measured in maternal serum samples using mass spectrometry methods and averaged if more than one sample was available.

*Statistical analysis*. We compared geometric mean EDC concentrations in women with (*n* = 194–222) and without (*n* = 130–166) at least one follow-up visit when their children were 4 or 5 years of age. Then we calculated percentiles of EDC concentrations and compared median concentrations with medians in a nationally representative sample of U.S. women participating in the 2003–2004 National Health and Nutrition Examination Survey (NHANES) (CDC 2009; Patterson et al. 2009; SjÖdin et al. 2008). We also examined SRS scores according to the above listed covariates.

We implemented a two-stage semi-Bayesian model that has previously been used to examine multiple environmental pollutants in relation to human health (De Roos et al. 2001; Kalkbrenner et al. 2010). The semi-Bayesian model controls for co-pollutant and traditional confounders, addresses multiple comparisons by reducing the influence of outlying estimates, and improves the precision and plausibility of estimates by shrinking the beta coefficients toward the mean of their exchangeability group as a function of their precision and a prespecified normally distributed residual variance parameter (τ^2^). This technique allows individual beta parameters to borrow information from other betas in the same exchangeability group (Greenland 1994; Greenland and Poole 1994). In the first stage of the model, SRS scores were regressed on all 52 EDC biomarkers and the above-mentioned confounders in a single linear mixed model to account for the repeated SRS measures. In the second stage, the beta parameters from this model were regressed against an exchangeability matrix, their covariance, and τ^2^.

Exchangeability predictors were chosen based on our *a priori* expectation that structurally similar chemicals would share common mechanisms. Our exchangeability matrix included indicator variables (0/1) for dibutyl phthalate (DBP) metabolites [mono-*n*-butyl phthalate (MBP) and monoisobutyl phthalate (MiBP)], di(2-ethylhexyl) phthalate (DEHP) metabolites [mono(2-ethylhexyl) phthalate (MEHP), MEHHP, and mono(2-ethyl-5-carboxypentyl) phthalate (MECPP)], BFRs, PFASs, PCBs, OC pesticides, and whether the chemical was persistent or nonpersistent (persistent chemicals were measured in serum; nonpersistent chemicals were measured in urine) (see Supplemental Material, Table S2). We specified a τ^2^ that assumed beta parameters would be between –10 and 10 (i.e., within 1 SD of the mean SRS score in the normative sample). A smaller τ^2^ implies that most of the association is explained by factors specified in the exchangeability matrix, whereas a larger τ^2^ assumes that factors not specified in the exchangeability matrix explain more of the association. First-stage models were fit using SAS PROC MIXED and second stage models were fit with SAS PROC IML code (SAS Institute Inc., Cary, NC, USA) (Witte et al. 1998).

*Sensitivity analyses*. We conducted additional analyses to test the robustness of our results. First, we used inverse probability weights to account for nonrandom censoring due to loss to follow-up (Cole and Hernan 2008). Second, we specified a smaller τ^2^ parameter in the second stage of our semi-Bayesian regression model that corresponded to a 10-point range of SRS scores (i.e., within 0.5 SD of the mean SRS scores in the normative sample). Third, we tested the assumption of a fixed residual variance by conducting an empirical Bayesian analysis (i.e., with τ^2^ = 0 and the residual variance estimated from the observed data). Finally, we examined the relationship between individual chemicals concentrations and SRS scores without restricting to mother–child pairs with complete EDC biomarker data.

*Exploratory analysis*. We investigated whether the associations between EDC biomarkers and SRS scores were modified by child sex using confounder-adjusted single-pollutant models with a product interaction term between child sex and biomarker concentrations to produce sex-specific estimates.

## Results

Among 389 women who delivered singleton infants, 222 mother–child pairs (57%) completed at least one follow-up visit at 4 (*n* = 184) or 5 (*n* = 205) years of age. Our analyses included 175 (45%) mother–child pairs with 310 observations at 4 and 5 years of age after excluding those who were missing data for confounders (*n* = 13), EDC biomarkers (*n* = 33), or both (*n* = 1). One hundred thirty-five children had two SRS measures at both 4 and 5 years of age. Average urine and serum biomarker concentrations were similar among women with and without follow-up when their child was 4 or 5 years old (see Supplemental Material, Table S3).

Individual women had 21–52 (median, 44) detectable EDCs in their serum or urine during pregnancy. Median EDC concentrations in HOME study women were similar to those of adult women in the NHANES 2003–2004 (Table 1).

Repeated SRS total *T*-scores in individual children at 4 and 5 years were highly correlated, with an intraclass correlation coefficient of 0.74. Higher SRS scores, indicating more autistic behaviors, were observed among children whose mothers were socioeconomically disadvantaged or exposed to tobacco smoke during pregnancy (Table 2).

**Table 2:** SRS total T-scores among 4- and 5-year-old children in Cincinnati, Ohio, according to demographic, perinatal, and environmental factors.

Demographic, perinatal, or environmental factor	*n* (%)	Total *T*-score (mean ± SD)^*a*^
Overall	175	51 ± 9
Maternal race
White	117 (67)	48 ± 7
Black	50 (29)	58 ± 12
Other	8 (5)	45 ± 6
Maternal age (years)
< 25	36 (21)	57 ± 13
25 to < 35	115 (66)	50 ± 8
≥ 35	24 (14)	47 ± 7
Maternal education
Graduate/professional school	92 (53)	47 ± 6
Some college	48 (27)	53 ± 9
High school	21 (12)	57 ± 13
< High school	14 (8)	60 ± 11
Marital status
Married	122 (70)	48 ± 6
Unmarried, living together	15 (9)	55 ± 10
Unmarried, living alone	38 (22)	59 ± 12
Annual household income
≥ $80,000	46 (26)	47 ± 5
$40,000 to < 80,000	66 (38)	48 ± 6
$20,000 to < 40,000	26 (15)	54 ± 8
< $20,000	37 (21)	60 ± 13
Maternal depressive symptoms
Minimal	148 (85)	50 ± 8
Mild	16 (9)	58 ± 10
Moderate/severe	11 (6)	59 ± 14
Maternal IQ
1st tertile (58–101)	58 (33)	58 ± 11
2nd tertile (> 101–114)	61 (35)	49 ± 7
3rd tertile (115–134)	56 (32)	47 ± 6
Child sex
Girls	95 (54)	52 ± 10
Boys	80 (46)	49 ± 8
Caregiving environment score
Low	30 (17)	58 ± 9
Medium	27 (15)	58 ± 13
High	118 (67)	48 ± 6
Maternal serum cotinine concentration (ng/mL)
No exposure (< 0.015)	68 (39)	48 ± 6
Secondhand exposure (0.015–3)	94 (54)	53 ± 11
Active exposure (> 3)	13 (7)	55 ± 10
Prenatal vitamin use frequency
Daily	132 (75)	49 ± 7
1–6 times/week	17 (10)	52 ± 12
Never or few times/month	26 (15)	58 ± 13
Maternal employment
None	30 (17)	56 ± 14
Any	145 (83)	50 ± 8
Parity
Nulliparous	80 (46)	50 ± 9
1–2	83 (47)	51 ± 10
≥ 3	12 (7)	58 ± 11
Maternal insurance source
Private	129 (74)	48 ± 7
Public/none	46 (26)	58 ± 12
^***a***^Higher scores indicate more autistic behaviors.		

In single-pollutant models, the extent of confounding related to SES or perinatal/caregiving/maternal factors depended on the chemical class; in some cases point estimates were attenuated down and toward the null [e.g., polybrominated diphenyl ethers (PBDEs)] and in other cases up and through the null (e.g., PCBs and OC pesticides) (see Supplemental Material, Table S4).

**Figure 1:**
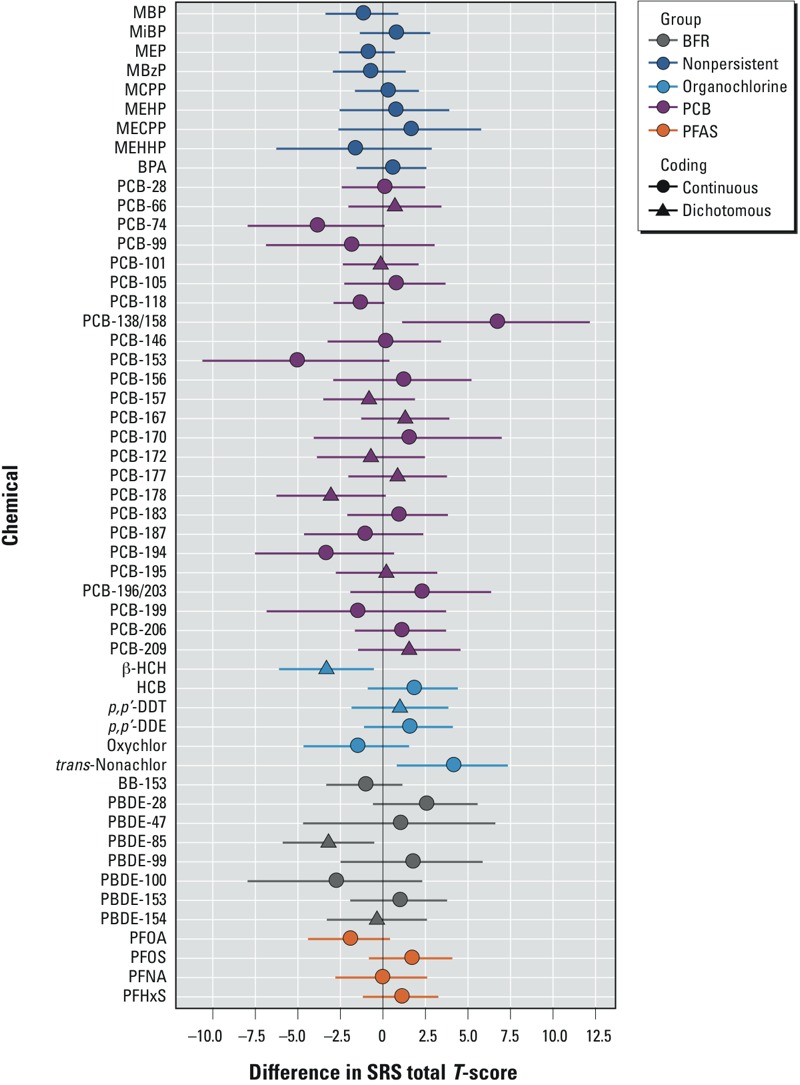
Associations between maternal gestational urine or serum EDC concentrations and SRS total T-scores in 4- and 5-year-old Cincinnati children using a semi-Bayesian model (*n* = 175). PCB-66, PCB‑101, PCB-157, PCB-167, PCB-172, PCB-177, PCB-178, PCB-195, PCB-209, β-HCH, DDT, PBDE-85, PBDE-154, and PBDE‑183 are coded as detected vs. nondetectable. The displayed betas are the change in SRS scores among children born to women with detectable vs. nondetectable levels of these chemicals. All other chemicals were treated as continuous log_10_-transformed variables that are divided by two times their standard deviation to put them on a comparable scale to the dichotomous variables. Adjusted for demographic factors for the covariate-adjusted semi-Bayesian model (see Table 2), as well as depressive symptoms during pregnancy (continuous), HOME score (continuous), and gestational serum cotinine concentration (continuous log10 transformed). The residual variance (τ^2^) of the semi-Bayesian model was set to 26.03 (20-point range). The exchangeability matrix is contains an intercept and indicator (0/1) variables for OCs, PCBs, BFRs, PFASs, DEHP metabolites, DBP metabolites, and persistent vs. nonpersistent chemicals. Whiskers indicate 95% CIs.

After adjustment for confounders and all EDC exposures in our semi-Bayesian hierarchical regression model, most EDC concentrations were associated with negligible changes in SRS scores, with the absolute change being ≤ 1.5 point (Figure 1; also see Supplemental Material, Table S4). Many EDCs showed very imprecise associations with SRS scores, as indicated by the larger SEs (> 2) and wide confidence intervals (e.g., PCB-153, PCB-199, and PBDE-100). PCB-138/158 and PCB-153 had the largest associations with SRS scores, but were very imprecise because of their high correlations with each other (Pearson *r* = 0.89) and other PCBs. Below we highlight some chemicals associated with ≥ 1.5-point change in SRS scores that were reasonably precise (SEs < 2.0). Almost all of the stronger and precise associations were observed for persistent chemicals, except for the two oxidative DEHP metabolites.

At least one chemical from each class of EDCs we examined was associated with higher SRS scores, which are consistent with more autistic behaviors. The one exception was the phenol BPA, which was not associated with SRS scores. Notably, maternal serum concentrations of *trans*-nonachlor and PBDE-28 were associated with higher SRS scores (β = 4.1; 95% CI: 0.8, 7.3 and β = 2.5; 95% CI: –0.6, 5.6) with each 2-SD increase in concentration.

Lower SRS scores, consistent with less autistic behaviors, were associated with prenatal exposure to at least one chemical from each class of EDCs. Detectable (vs. nondetectable) serum concentrations of PCB-178 (*n* = 27; β = –3.0; 95% CI: –6.3, 0.2), β-hexachlorocyclohexane (β-HCH) (*n* = 52; β = –3.3; 95% CI: –6.1, –0.5), or PBDE-85 (*n* = 86; β = –3.1; 95% CI: –5.9 –0.5) were associated with lower SRS scores. Each 2-SD increase in serum perfluorooctanoate (PFOA) (β = –2.0; 95% CI: –4.4, 0.4) was associated with SRS scores.

*Sensitivity analyses*. The patterns of our results were similar when we accounted for censoring due to loss to follow-up, used a smaller τ^2^ in our semi-Bayesian model, or used an empirical Bayes analysis (see Supplemental Material, Table S5). Most of the confounder-adjusted single-pollutant model results were similar and more precise because of the increased sample size (*n* = 181–208) when we did not restrict to mother–child pairs with complete EDC biomarker data (see Supplemental Material, Table S5). However, the associations between perfluorooctane sulfonate (PFOS) or perfluorononanoate (PFNA) and SRS scores were attenuated toward the null when we included these additional women.

**Figure 2:**
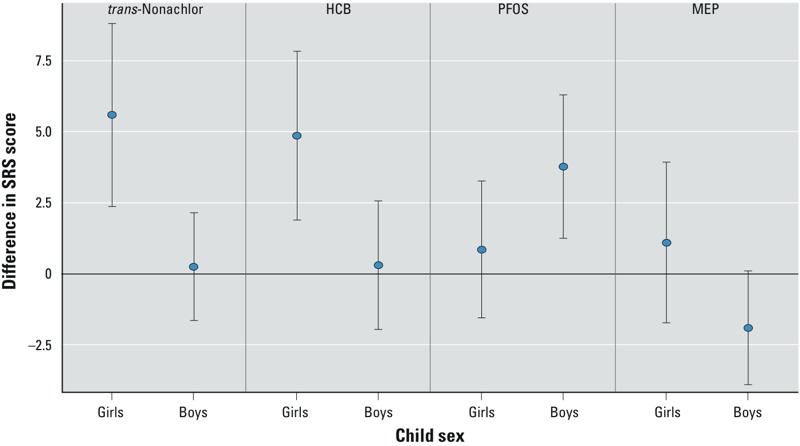
Confounder-adjusted associations between maternal gestational urinary or serum EDC concentrations and SRS total T-scores in 4- and 5-year-old Cincinnati children, stratified by child sex (*n* = 175). All displayed betas are the change in child SRS scores with a 2‑SD increase in log_10_-transformed maternal chemical concentration. Adjusted for demographic factors (see Table 2) as well as depressive symptoms during pregnancy (continuous), HOME score (continuous), and gestational serum cotinine concentration (continuous log_10_ transformed). Whiskers indicate 95% CIs. Only associations with significant chemical × sex interactions are displayed (p < 0.10).

*Exploratory analysis*. With few exceptions (Figure 2; see also Supplemental Material, Table S6), most EDC biomarker and SRS score associations were similar for boys and girls (interaction *p*-values > 0.10) (see Supplemental Material, Table S5). However, a 2-SD increase in serum hexachlorobenzene (HCB) (interaction *p*-value = 0.02) and *trans*-nonachlor (interaction *p*-value < 0.01) was positively associated with higher SRS scores in girls (HCB β = 4.9; 95% CI: 1.9, 7.8; *trans*-nonachlor β = 5.6; 95% CI: 2.4, 8.8), but not in boys (HCB β = 0.3; 95% CI: –1.9, 2.6; *trans*-nonachlor β = 0.3; 95% CI: –1.7, 2.2). Serum PFOS concentrations were positively associated with SRS scores in in boys (β = 3.8; 95% CI: 1.3, 6.3), but not girls (β = 0.9; 95% CI: –1.5, 3.3) (interaction *p*-value = 0.08). A 2-SD increase in monoethyl phthalate (MEP) concentrations was associated with lower SRS scores (consistent with fewer autistic behaviors) in boys (β = –1.9; 95% CI: –3.9, 0.1), but not girls (β = 1.1; 95% CI: –1.7, 3.9) (interaction *p*-value = 0.09).

## Discussion

This work builds on the theory that ASDs are a spectrum of disorders with prenatal origins, where both genetic and environmental factors contribute to atypical neurodevelopment, resulting in more autistic behaviors, and, at the extreme end, clinical diagnosis. EDCs deserve consideration as candidate risk factors for ASDs because of their potential to alter hormonal axis functions that play an important role in neurodevelopment. Building on this, we employed a statistically rigorous design to screen 52 different candidate EDCs and identify those worth additional study. Most of the EDC biomarkers we examined were associated with modest and imprecise differences in SRS scores in children, including most of the PCBs, all of the phthalate metabolites, and BPA. Maternal serum *trans*-nonachlor and PBDE-28 concentrations were positively associated with more autistic behaviors, whereas PBDE-85, PCB-178, β-HCH, and PFOA concentrations were associated with less autistic behaviors.

Because there are few prior studies examining the link between EDC exposures and the risk of ASDs or autistic behaviors, we discuss studies examining early-life EDC exposures and other neurobehavioral traits or disorders below [e.g, attention deficit/hyperactivity disorder (ADHD)]. These comparisons are made assuming that EDCs may affect multiple neurobehavioral domains, or that neurodevelopmental disorders share common behavioral features. Study results may also vary because of differences in methods of assessing neurobehavior or ascertaining ASD cases, mechanisms underlying each chemical–neurobehavior association, study designs, the timing or method of exposure assessment, and statistical methods.

Prior studies suggest that prenatal BPA or phthalate exposures may be associated ADHD, anxiety, or autistic behaviors (Braun et al. 2011; Engel et al. 2010; Miodovnik et al. 2011). Using a prospective birth cohort of 137 mothers and their children, Miodovnik et al. (2011) reported that maternal urinary MEP and possibly BPA concentrations during pregnancy were associated with higher SRS scores in 7- to 9-year-old children. We observed an inverse association between MEP levels and SRS scores, and a null association for BPA. Even when we did not adjust for co-pollutant confounding, we observed an inverse association between MEP levels and SRS scores. The relatively high within-person variability of urinary MEP and BPA concentrations and different timing of urine collection across studies may account for different results. If the timing of exposure affects neurodevelopment, then future epidemiological studies trying to identify windows of susceptibility to nonpersistent chemicals, such as BPA and phthalates, will need to find ways to minimize exposure measurement error (Braun et al. 2009, 2012; Gioiosa et al. 2013).

Several studies have reported associations of *in utero* PCB exposure with neurobehavioral features of ADHD (Eubig et al. 2010; Sagiv et al. 2010), but few have examined ASD or autistic behaviors. A small nested case–control (*n* = 75 cases, *n* = 75 controls) study reported that higher maternal serum PCB and dichlorodiphenyldichloroethylene (DDE) concentrations were associated with increased odds of ASD diagnosis (Cheslack-Postava et al. 2013). We observed different patterns of association between individual PCB congeners and SRS scores. Consistent with their findings (Cheslack-Postava et al. 2013), we did observe a positive association between maternal DDE levels and SRS scores (β = 1.5; CI: –1.1, 4.1). There was no *a priori* reason to suspect that PCB-178 would be protective, and the neurotoxicity of individual PCB congeners has not been well investigated because many prior investigations have used individual or the sum of PCB congeners to assess this exposure mixture.

Using a retrospective cohort (*n* = 465 cases, *n* = 6,975 controls), Roberts et al. (2007) reported increased odds of ASD diagnosis among children whose mothers resided near agricultural fields sprayed with the OC pesticides dicofol and endosulfan during their pregnancies. Despite the widespread use of *trans*-nonachlor as an insecticide for citrus and corn crops from the 1960s to late 1980s, the neurotoxicity of *trans*-nonachlor, the related chlordanes, and HCB is relatively unstudied. We report that *trans*-nonachlor concentrations were associated with higher SRS scores, especially among girls. Consistent with this, a rodent study found that gestational chlordane exposure caused decreased testosterone concentrations and improved maze performance in female offspring (Cassidy et al. 1994).

Two prospective birth cohort studies (*n* = 152, *n* = 323) have reported associations between gestational PBDE exposure and ADHD-like behaviors, poorer mental and physical development, and lower IQ among children (Eskenazi et al. 2013; Herbstman et al. 2010). Similar to the negative association between PBDE-85 concentrations and SRS scores we observed, a case–control study of 50 ASD cases and 25 controls reported that higher childhood serum PBDE-85 concentrations were associated with decreased odds of ASD diagnosis (Hertz-Picciotto et al. 2011). Another case–control study examining postmortem PBDE and PCB brain tissue concentrations in idiopathic ASD cases and controls was not consistent with our results (Mitchell et al. 2012).

The positive and negative associations between SRS scores and PBDE-28 and PBDE-85, respectively, may reflect congener-specific effects on different endocrine pathways. *In vitro* experiments show that tri-brominated PBDE-28 strongly potentiates triiodothyronine responses, but is a weaker antagonist of androgen and progesterone receptors than some pentabrominated PBDEs, such as PBDE-85. (Hamers et al. 2006). Additional experimental and epidemiological studies are necessary to identify potential mechanisms of PBDE action and confirm our findings (Dingemans et al. 2011).

Similar to the protective association we observed for PFOA, a cross-sectional study reported that PFAS exposures were associated with reduced prevalence of adult cognitive limitations (Power et al. 2013), and a prospective study of 320 children observed better cognitive abilities among children with higher prenatal PFOA exposure (Stein et al. 2013). In contrast, a cross-sectional study of U.S. adolescents found that higher PFOA and PFOS concentrations were associated with parent-reported ADHD (Hoffman et al. 2010). *In vitro* studies report that PFOA and PFOS are partial agonists of the human peroxisome proliferator–activated receptor-γ, and activation of this receptor may be neuroprotective (Kapadia et al. 2008). Contradictory results across studies may be attributable to the different neurobehavioral domains assessed and differences in the timing of exposure assessment (e.g., prenatal vs. childhood) in these studies.

The most notable associations in our study were for persistent chemicals with biological half-lives on the order of years (Geyer et al. 2004; Olsen et al. 2007; Wolff et al. 2000). The association between autistic behaviors and nonpersistent compounds, such as phthalates and BPA, may be attenuated toward the null because these chemicals are subject to more nondifferential exposure misclassification than persistent chemicals.

The SRS and other continuous measures of autistic behaviors have several desirable features for prospective cohorts such as this one. Although the SRS is not equivalent to clinical diagnosis, SRS scores provide a ranking of children’s autistic behaviors on a continuum and enhance statistical power (Bellinger 2004; Jones and Lord 2013). Studies using clinical diagnosis are necessary to determine whether environmental exposures associated with subtle changes in continuous distributions of autistic behavior increase the risk of ASDs. Finally, continuous measures may detect earlier or subclinical manifestations of ASD that clinical diagnoses cannot identify, but these symptoms may also be correlates of other behavioral disorders, including ADHD (Reiersen et al. 2007).

Our longitudinal study design had numerous strengths for identifying potential chemical risk factors for ASDs. We used sensitive and specific biomarkers of 52 unique EDC exposures during the developmentally sensitive *in utero* period and repeated assessments of autistic behaviors using a valid and reliable instrument (Bolte et al. 2008). In addition, we adjusted for numerous potential confounders, including gestational tobacco smoke exposure, socioeconomic factors, perinatal factors, caregiving environment, maternal IQ, and maternal depressive symptoms. Such comprehensive data collection comes at the cost of sample size, and though we increased statistical power by using repeated continuous outcome measures, our precision was modest. Residual confounding and nonrandom loss to follow-up is always a concern for observational longitudinal epidemiology studies. Although we controlled for both traditional covariates and EDC co-pollutants, the measured associations could be attributable to residual confounding. Nonrandom attrition could also bias our results in an unpredictable fashion, but our sensitivity analyses accounting for loss to follow-up did not suggest this was the case.

Failure to consider co-pollutant confounding may lead to erroneous inferences and ultimately ineffective public health interventions. By adjusting for multiple EDC exposures in a semi-Bayesian model, we accounted for co-pollutant confounding, let individual beta coefficients borrow information from their exchangeability group, and were able to compare the magnitude of association across chemicals. Although this is a considerable strength and is not typically done in epidemiological studies, it does risk unnecessary adjustment for chemicals that are not correlated with each other (Schisterman et al. 2009). In addition, we focused specifically on suspected EDCs that were measured in this study based on our *a priori* hypothesis, and did not account for other neurotoxicants such as lead and organophosphate pesticides.

We attempted to reduce type 1 errors and address multiple comparisons by using a semi-Bayesian model, but we may have failed to detect associations (i.e., type 2 errors) because of our modest sample size and the potential for misclassification of nonpersistent chemical exposures. Strict interpretation of *p*-values (e.g., *p* < 0.05) is not used in the Bayesian approach, so we focused on the relative rank of the magnitude and precision of the EDC effect estimates.

Future studies using semi-Bayesian models could incorporate discrete or continuous aspects of a chemical’s toxicity into their analysis, such as aryl hydrocarbon receptor binding affinity or potency to reduce androgen production (Howdeshell et al. 2008; Van den Berg et al. 2006). Other hormonal and molecular pathways susceptible to EDC disruption and relevant to ASD etiology, including oxytocin and vasopressin, also deserve consideration (Hammock and Young 2006; Wolstenholme et al. 2012). Finally, future work should consider the sum of the effect of EDCs; however, this will require additional toxicity data related to the biological activity of these chemicals in pathways related to ASDs. The imprecise associations we observed for many PCBs and the two DEHP oxidative metabolites shows that the high correlation between some chemicals makes it difficult, if not impossible, to disentangle individual associations of highly correlated exposures. Future studies may consider summing or weighting these according to their biological activity (Safe 1998).

Most biomarkers of maternal EDC exposure during pregnancy were not clearly associated with autistic behaviors in 4- and 5-year-old children in this cohort. Although this hypothesis-generating study cannot definitely identify chemical risk factors for ASDs, these results suggest that additional studies examining the relationship between ASDs and gestational exposure to PFOA, PCB-178, PBDE-28, PBDE-85, β-HCH, and *trans*-nonachlor are warranted. Given the mixture of environmental chemicals that pregnant women are exposed to, future studies should consider statistical techniques that account for the complex mixture of potentially modifiable prenatal environmental chemical exposures that might be associated with ASDs.

## Supplemental Material

(598 KB) PDF
