# Supplementary material for: Gestational Exposure to Endocrine-Disrupting Chemicals and Reciprocal Social, Repetitive, and Stereotypic Behaviors in 4- and 5-Year-Old Children: The HOME Study
Source: Environ Health Perspect. 2014 Mar 12;122(5):513–20. doi: 10.1289/ehp.1307261 (PMC4014765; doi:10.1289/ehp.1307261)
Supplement: (598 KB) PDF [file ehp.1307261.s001.pdf]

Environmental Health Perspectives doi:10.1289/ehp.1307261

Supplemental Material

Gestational Exposure to Endocrine-Disrupting Chemicals and Reciprocal Social, Repetitive, and Stereotypic Behaviors in 4- and 5-Year-Old Children: The HOME Study

Joseph M. Braun, Amy E. Kalkbrenner, Allan C. Just, Kimberly Yolton, Antonia M. Calafat, Andreas Sjödin, Russ Hauser, Glenys M. Webster, Aimin Chen, and Bruce P. Lanphear

Table of Contents **Page**

Table S1. List of environmental chemicals or metabolites analyzed in HOME Study samples and reasons for exclusion from final analysis 2

Table S2. Specification of exchangeability matrix in semi-Bayesian regression model. 5

Table S3. Endocrine disrupting chemical concentrations among mothers who did and did not complete at least one follow-up visit at 4-5 years of age 7

Table S4. Unadjusted and covariate adjusted associations between SRS Total T score in 4 and 5 year old Cincinnati children and maternal gestational urinary or serum endocrine disrupting chemical concentrations 9

Table S5. Covariate adjusted, multi-pollutant models of endocrine disrupting chemicals and SRS Total T scores: Assessing model sensitivity 12

Table S6. Adjusted sex-specific change in SRS Total T scores in 4 and 5 year old Cincinnati children with increasing maternal gestational urinary or serum endocrine disrupting chemical concentrations 16

Table S1. List of environmental chemicals or metabolites analyzed in HOME Study samples and reasons for exclusion from final analysis.

Chemical or Metabolite Name	Abbreviation	Chemical Class	Included in Analysis	Reason for Exclusion
Mono-n-butyl phthalate	MBP	Phthalate	Yes	
Mono-i-butyl phthalate	MiBP	Phthalate	Yes	
Mono-ethyl phthalate	MEP	Phthalate	Yes	
Mono-benzyl phthalate	MBzP	Phthalate	Yes	
Mono-3-carboxypropyl phthalate	MCPP	Phthalate	Yes	
Mono-2-ethyl-hexyl phthalate	MEHP	Phthalate	Yes	
Mono-2-ethyl-5-oxohexyl phthalate	MEOHP	Phthalate	No	Pearson R of 0.99 with MEHHP
Mono-2-ethyl-5-hydroxyhexyl phthalate	MEHHP	Phthalate	Yes	
Mono-2-ethyl-5-carboxypentyl phthalate	MECPP	Phthalate	Yes	
Bisphenol A	BPA	Phenol	Yes	
Polychlorinated biphenyl-18	PCB-18	Polychlorinated biphenyl	No	Frequency of detection < 20%
Polychlorinated biphenyl-28	PCB-28	Polychlorinated biphenyl	Yes	
Polychlorinated biphenyl-44	PCB-44	Polychlorinated biphenyl	No	Frequency of detection < 20%
Polychlorinated biphenyl-49	PCB-49	Polychlorinated biphenyl	No	Frequency of detection < 20%
Polychlorinated biphenyl-52	PCB-52	Polychlorinated biphenyl	No	Frequency of detection < 20%
Polychlorinated biphenyl-66	PCB-66	Polychlorinated biphenyl	Yes	
Polychlorinated biphenyl-74	PCB-74	Polychlorinated biphenyl	Yes	
Polychlorinated biphenyl-87	PCB-87	Polychlorinated biphenyl	No	Frequency of detection < 20%
Polychlorinated biphenyl- 99	PCB-99	Polychlorinated biphenyl	Yes	
Polychlorinated biphenyl-101	PCB-101	Polychlorinated biphenyl	Yes	
Polychlorinated biphenyl-105	PCB-105	Polychlorinated biphenyl	Yes	
Polychlorinated biphenyl-110	PCB-110	Polychlorinated biphenyl	No	Frequency of detection < 20%
Polychlorinated biphenyl-118	PCB-118	Polychlorinated biphenyl	Yes	
Polychlorinated biphenyl-128	PCB-128	Polychlorinated biphenyl	No	Frequency of detection < 20%
Polychlorinated biphenyl-138/158	PCB-138/158	Polychlorinated biphenyl	Yes	
Polychlorinated biphenyl-146	PCB-146	Polychlorinated biphenyl	Yes	

Chemical or Metabolite Name	Abbreviation	Chemical Class	Included in Analysis	Reason for Exclusion
Polychlorinated biphenyl-149	PCB-149	Polychlorinated biphenyl	No	Frequency of detection < 20%
Polychlorinated biphenyl-151	PCB-151	Polychlorinated biphenyl	No	Frequency of detection < 20%
Polychlorinated biphenyl-153	PCB-153	Polychlorinated biphenyl	Yes	
Polychlorinated biphenyl-156	PCB-156	Polychlorinated biphenyl	Yes	
Polychlorinated biphenyl-157	PCB-157	Polychlorinated biphenyl	Yes	
Polychlorinated biphenyl-167	PCB-167	Polychlorinated biphenyl	Yes	
Polychlorinated biphenyl -170	PCB-170	Polychlorinated biphenyl	Yes	
Polychlorinated biphenyl -172	PCB-172	Polychlorinated biphenyl	Yes	
Polychlorinated biphenyl -177	PCB-177	Polychlorinated biphenyl	Yes	
Polychlorinated biphenyl -178	PCB-178	Polychlorinated biphenyl	Yes	
Polychlorinated biphenyl -180	PCB-180	Polychlorinated biphenyl	No	Pearson R of 0.96 with PCB-170
Polychlorinated biphenyl -183	PCB-183	Polychlorinated biphenyl	Yes	
Polychlorinated biphenyl -187	PCB-187	Polychlorinated biphenyl	Yes	
Polychlorinated biphenyl -189	PCB-189	Polychlorinated biphenyl	No	Frequency of detection < 20%
Polychlorinated biphenyl -194	PCB-194	Polychlorinated biphenyl	Yes	
Polychlorinated biphenyl -195	PCB-195	Polychlorinated biphenyl	Yes	
Polychlorinated biphenyl -196/203	PCB-196/203	Polychlorinated biphenyl	Yes	
Polychlorinated biphenyl -199	PCB-199	Polychlorinated biphenyl	Yes	
Polychlorinated biphenyl -206	PCB-206	Polychlorinated biphenyl	Yes	
Polychlorinated biphenyl -209	PCB-209	Polychlorinated biphenyl	Yes	
β-Hexachlorocyclohexane	β-HCH	Organochlorine pesticide	Yes	
γ-Hexachlorocyclohexane	γ-HCH	Organochlorine pesticide	No	Frequency of detection < 20%
Hexachlorobenzene	HCB	Organochlorine pesticide	Yes	
Mirex		Organochlorine pesticide	No	Frequency of detection < 20%
p'p'-Dichlorodiphenyltrichloroethane	PP-DDT	Organochlorine pesticide	Yes	
o'p'-Dichlorodiphenyltrichloroethane	OP-DDT	Organochlorine pesticide	No	Frequency of detection < 20%
p'p'-Dichlorodiphenyldichloroethylene	PP-DDE	Organochlorine pesticide	Yes	
Oxychlordane		Organochlorine pesticide	Yes	

**Chemical or Metabolite Name**	**Abbreviation**	**Chemical Class**	**Included in Analysis**	**Reason for Exclusion**
trans-Nonachlor		Organochlorine pesticide	Yes	
Brominated biphenyl-153	BB-153	Brominated flame retardant	Yes	
Polybrominated diphenyl ether-17	PBDE-17	Brominated flame retardant	No	Frequency of detection < 20%
Polybrominated diphenyl ether-28	PBDE-28	Brominated flame retardant	Yes	
Polybrominated diphenyl ether-47	PBDE-47	Brominated flame retardant	Yes	
Polybrominated diphenyl ether-66	PBDE-66	Brominated flame retardant	No	Frequency of detection < 20%
Polybrominated diphenyl ether-85	PBDE-85	Brominated flame retardant	Yes	
Polybrominated diphenyl ether-99	PBDE-99	Brominated flame retardant	Yes	
Polybrominated diphenyl ether-100	PBDE-100	Brominated flame retardant	Yes	
Polybrominated diphenyl ether-153	PBDE-153	Brominated flame retardant	Yes	
Polybrominated diphenyl ether-154	PBDE-154	Brominated flame retardant	Yes	
Polybrominated diphenyl ether-183	PBDE-183	Brominated flame retardant	Yes	Frequency of detection < 20%
Perfluorooctanoate	PFOA	Perfluorinated chemical	Yes	
Perfluorooctane sulfate	PFOS	Perfluorinated chemical	Yes	
Perfluorononanoate	PFNA	Perfluorinated chemical	Yes	
Perfluorohexane sulfonate	PFHxS	Perfluorinated chemical	Yes	

Table S2. Specification of exchangeability matrix in semi-Bayesian regression model.

Chemical	Di-butyl phthalate metabolite	Di-2-ethylhexyl phthalate metabolite	Polychlorinated biphenyl	Organochlorine Pesticide	Polybrominated diphenyl ether	Perfluoralkyl Substance	Persistent Chemical
MBP	1	0	0	0	0	0	0
MiBP	1	0	0	0	0	0	0
MEP	0	0	0	0	0	0	0
MBzP	0	0	0	0	0	0	0
MCPP	0	0	0	0	0	0	0
MEHP	0	1	0	0	0	0	0
MECPP	0	1	0	0	0	0	0
MEHHP	0	1	0	0	0	0	0
BPA	0	0	0	0	0	0	0
PCB 28	0	0	1	0	0	0	1
PCB 66	0	0	1	0	0	0	1
PCB 74	0	0	1	0	0	0	1
PCB 99	0	0	1	0	0	0	1
PCB 101	0	0	1	0	0	0	1
PCB 105	0	0	1	0	0	0	1
PCB 118	0	0	1	0	0	0	1
PCB 138/158	0	0	1	0	0	0	1
PCB 146	0	0	1	0	0	0	1
PCB 153	0	0	1	0	0	0	1
PCB 156	0	0	1	0	0	0	1
PCB 157	0	0	1	0	0	0	1
PCB 167	0	0	1	0	0	0	1
PCB 170	0	0	1	0	0	0	1
PCB 172	0	0	1	0	0	0	1
PCB 177	0	0	1	0	0	0	1
PCB 178	0	0	1	0	0	0	1
PCB 183	0	0	1	0	0	0	1

Chemical	Di-butyl phthalate metabolite	Di-2-ethylhexyl phthalate metabolite	Polychlorinated biphenyl	Organochlorine Pesticide	Polybrominated diphenyl ether	Perfluoralkyl Substance	Persistent Chemical
PCB 187	0	0	1	0	0	0	1
PCB 194	0	0	1	0	0	0	1
PCB 195	0	0	1	0	0	0	1
PCB 196/203	0	0	1	0	0	0	1
PCB 199	0	0	1	0	0	0	1
PCB 206	0	0	1	0	0	0	1
PCB 209	0	0	1	0	0	0	1
β-HCH	0	0	0	1	0	0	1
HCB	0	0	0	1	0	0	1
p′p′-DDT	0	0	0	1	0	0	1
p′p′-DDE	0	0	0	1	0	0	1
Oxychlordane	0	0	0	1	0	0	1
trans-Nonachlor	0	0	0	1	0	0	1
BB 153	0	0	0	0	0	0	1
PBDE 28	0	0	0	0	1	0	1
PBDE 47	0	0	0	0	1	0	1
PBDE 85	0	0	0	0	1	0	1
PBDE 99	0	0	0	0	1	0	1
PBDE 100	0	0	0	0	1	0	1
PBDE 153	0	0	0	0	1	0	1
PBDE 154	0	0	0	0	1	0	1
PFOA	0	0	0	0	0	1	1
PFOS	0	0	0	0	0	1	1
PFNA	0	0	0	0	0	1	1
PFHxS	0	0	0	0	0	1	1

Table S3. Endocrine disrupting chemical concentrations among mothers who did and did not complete at least one follow-up visit at 4-5 years of age.

Chemical	No. with follow-up	With follow-up: Median (25th, 75th)	No. without follow-up	No follow-up: Median (25th, 75th)
MBP	222	25.5 (17.1, 36.6)	165	24.3 (15.8, 41.6)
MiBP	222	5.2 (3.0, 7.9)	165	4.6 (3.1, 7.1)
MEP	222	126.4 (66.6, 278.0)	165	139.3 (72.0, 285.3)
MBzP	222	9.7 (5.4, 16.4)	165	8.3 (5.2, 14.6)
MCPP	222	2.3 (1.6, 3.3)	165	2.1 (1.6, 3.2)
MEHP	222	4.4 (3.0, 8.4)	165	4.7 (2.6, 10.1)
MECPP	222	32.9 (21.3, 65.6)	165	33.3 (21.2, 61.1)
MEHHP	222	21.9 (14.3, 48.1)	165	24.9 (14.8, 42.4)
BPA	222	2.0 (1.4, 3.1)	166	1.9 (1.3, 2.7)
PCB 28	194	1.0 (0.7, 1.6)	131	1.0 (0.4, 1.6)
PCB 66	194	0.6 (0.2, 1.0)	131	0.6 (0.1, 1.0)
PCB 74	204	2.8 (2.0, 4.3)	140	2.9 (1.9, 4.1)
PCB 99	194	2.8 (1.9, 3.9)	131	2.9 (1.8, 3.9)
PCB 101	194	0.1 (0.0, 0.5)	131	0.1 (0.0, 0.4)
PCB 105	194	1.1 (0.8, 1.7)	131	1.1 (0.8, 1.6)
PCB 118	209	5.0 (3.4, 7.4)	149	4.8 (3.3, 6.8)
PCB 138/158	209	7.8 (5.4, 11.6)	149	7.9 (5.4, 11.5)
PCB 146	194	1.1 (0.8, 1.7)	131	1.1 (0.6, 1.7)
PCB 153	210	11.2 (7.7, 15.9)	150	10.9 (7.3, 15.1)
PCB 156	194	1.6 (1.0, 2.5)	131	1.5 (0.9, 2.4)
PCB 157	194	0.2 (0.1, 0.6)	131	0.1 (0.0, 0.5)
PCB 167	194	0.5 (0.1, 0.6)	131	0.2 (0.1, 0.6)
PCB 170	202	2.9 (1.8, 4.3)	139	2.6 (1.7, 4.3)
PCB 172	194	0.1 (0.0, 0.4)	131	0.1 (0.0, 0.4)
PCB 177	194	0.5 (0.1, 0.7)	131	0.4 (0.1, 0.7)
PCB 178	194	0.2 (0.1, 0.7)	131	0.2 (0.1, 0.7)
PCB 183	194	1.0 (0.6, 1.4)	131	0.9 (0.5, 1.3)
PCB 187	194	2.1 (1.4, 3.3)	131	2.0 (1.4, 3.0)
PCB 194	194	1.4 (0.9, 2.2)	131	1.3 (0.8, 2.0)
PCB 195	194	0.1 (0.0, 0.5)	131	0.1 (0.0, 0.4)
PCB 196/203	194	1.6 (1.1, 2.6)	131	1.6 (1.0, 2.3)
PCB 199	194	1.3 (0.8, 2.2)	131	1.2 (0.7, 2.1)

**Chemical**	**No. with follow-up**	**With follow-up: Median (25th, 75th)**	**No. without follow-up**	**No follow-up: Median (25th, 75th)**
PCB 206	193	0.8 (0.6, 1.2)	131	0.9 (0.5, 1.4)
PCB 209	194	0.1 (0.0, 0.4)	131	0.1 (0.0, 0.5)
β−HCH	194	0.3 (0.2, 1.8)	131	0.3 (0.2, 1.4)
HCB	194	7.1 (5.6, 9.1)	131	7.3 (5.5, 9.8)
p′p′-DDT	194	1.9 (0.3, 3.2)	131	1.9 (0.3, 3.2)
p′p′-DDE	210	70.5 (54.8, 96.7)	150	79.1 (55.8, 106.0)
Oxychlordane	194	5.1 (3.4, 7.2)	131	5.6 (3.4, 8.5)
trans-Nonachlor	194	7.4 (5.1, 10.8)	131	8.0 (4.7, 12.9)
BB 153	194	1.1 (0.6, 1.9)	131	1.1 (0.5, 1.8)
PBDE 28	194	1.0 (0.5, 1.7)	131	0.9 (0.5, 1.7)
PBDE 47	209	18.6 (10.3, 33.8)	150	20.0 (12.0, 35.3)
PBDE 85	194	0.2 (0.0, 0.8)	131	0.4 (0.1, 0.9)
PBDE 99	202	4.3 (2.3, 7.6)	144	4.7 (2.6, 9.9)
PBDE 100	194	3.4 (2.0, 7.5)	131	4.4 (2.3, 8.2)
PBDE 153	194	4.2 (2.4, 9.0)	130	5.0 (2.9, 9.7)
PBDE 154	194	0.1 (0.0, 0.8)	131	0.3 (0.1, 0.9)
PFOA	206	5.5 (3.7, 7.5)	151	5.7 (3.9, 7.8)
PFOS	206	13.7 (9.3, 18.0)	151	13.7 (9.8, 18.1)
PFNA	206	0.9 (0.7, 1.2)	151	0.9 (0.7, 1.2)
PFHxS	205	1.5 (0.9, 2.4)	151	1.5 (0.9, 2.2)

Concentrations are displayed in units of ng/g lipds (PCBs, PBDEs, and OC pesticides), μg/g creatinine (phthalates and BPA) and μg/L (PFAS).

Table S4. Unadjusted and covariate adjusted associations (β [95% CI]) between SRS Total T score in 4 and 5 year old Cincinnati children and maternal gestational urinary or serum endocrine disrupting chemical concentrations.

Chemical	Unadjusted	Model 1: Adjusted for SES Variables[a]	Model 2: Adjusted for Perinatal, Maternal, and Caregiving Variables[b]	Model 3: Fully Adjust[c]	Model 4: Single Pollutant Models, No Restriction for Exposures
MBP	0.8 (-1.7, 3.3)	-0.7 (-2.6, 1.2)	-0.4 (-2.3, 1.5)	-0.4 (-2.2, 1.4)	-0.8 (-2.4, 0.9)
MiBP	1.3 (-0.9, 3.4)	-0.2 (-2.1, 1.6)	-0.1 (-2.0, 1.7)	0.3 (-1.5, 2.1)	0.1 (-1.6, 1.9)
MEP	1.3 (-1.1, 3.6)	0.2 (-1.6, 2.0)	-0.9 (-2.7, 1.0)	-0.5 (-2.2, 1.3)	-0.2 (-1.9, 1.5)
MBzP	2.4 (-0.2, 5.0)	-0.9 (-3.2, 1.4)	-0.5 (-2.6, 1.5)	-0.6 (-2.7, 1.5)	-0.7 (-2.5, 1.2)
MCPP	-0.4 (-3.0, 2.1)	1.2 (-1.0, 3.4)	0.7 (-1.3, 2.6)	1.1 (-0.9, 3.0)	0.5 (-1.4, 2.4)
MEHP	-0.7 (-3.5, 2.2)	1.5 (-0.6, 3.7)	0.8 (-1.1, 2.6)	1.4 (-0.4, 3.2)	1.1 (-0.7, 2.8)
MECPP	-0.3 (-3.0, 2.4)	1.9 (-0.2, 4.1)	1.1 (-0.8, 2.9)	1.6 (-0.3, 3.4)	1.3 (-0.4, 3.1)
MEHHP	-0.5 (-3.4, 2.4)	1.4 (-0.8, 3.6)	0.6 (-1.3, 2.6)	1.1 (-0.8, 3.0)	0.7 (-1.1, 2.5)
BPA	1.6 (-0.7, 3.8)	0.7 (-1.3, 2.8)	1.0 (-0.8, 2.8)	1.1 (-1.0, 3.1)	0.9 (-0.9, 2.8)
PCB 28	-0.4 (-2.5, 1.8)	2.0 (-0.2, 4.2)	0.7 (-1.0, 2.5)	1.0 (-0.9, 2.8)	1.0 (-0.8, 2.8)
PCB 66 [d]	0.3 (-2.4, 2.9)	1.8 (-0.7, 4.3)	0.8 (-1.5, 3.2)	0.9 (-1.4, 3.3)	1.1 (-1.2, 3.4)
PCB 74	-3.3 (-5.8, -0.8)	0.3 (-1.8, 2.5)	-0.7 (-2.6, 1.3)	-0.6 (-2.7, 1.6)	-0.2 (-2.3, 2.0)
PCB 99	-0.7 (-2.9, 1.5)	0.4 (-1.7, 2.6)	0.0 (-2.1, 2.0)	0.0 (-2.1, 2.1)	-0.2 (-2.3, 1.9)
PCB 101 [d]	1.2 (-1.9, 4.4)	0.4 (-2.0, 2.8)	0.1 (-2.0, 2.1)	-0.2 (-2.1, 1.8)	-0.1 (-2.1, 1.8)
PCB 105	0.7 (-1.5, 2.9)	1.2 (-0.9, 3.2)	0.4 (-1.8, 2.6)	0.5 (-1.7, 2.6)	0.3 (-1.8, 2.4)
PCB 118	-1.1 (-2.9, 0.8)	0.4 (-1.8, 2.6)	-0.5 (-2.4, 1.4)	-0.4 (-2.4, 1.6)	0.0 (-2.1, 2.0)
PCB 138/158	-2.5 (-5.1, 0.1)	0.0 (-2.2, 2.3)	-0.4 (-2.5, 1.8)	-0.2 (-2.5, 2.1)	-0.2 (-2.5, 2.1)
PCB 146	-3.5 (-5.7, -1.2)	-0.4 (-2.6, 1.9)	-1.0 (-3.5, 1.6)	-0.9 (-3.5, 1.6)	-1.0 (-3.6, 1.5)
PCB 153	-4.8 (-7.1, -2.6)	-1.0 (-3.4, 1.3)	-1.6 (-4.2, 1.0)	-1.5 (-4.1, 1.2)	-1.4 (-4.0, 1.2)
PCB 156	-5.2 (-7.9, -2.5)	-0.7 (-3.3, 1.9)	-1.3 (-4.2, 1.7)	-1.2 (-4.0, 1.6)	-1.2 (-4.0, 1.5)
PCB 157 [d]	-3.7 (-6.2, -1.2)	0.4 (-1.9, 2.6)	-0.8 (-2.8, 1.2)	-0.5 (-2.6, 1.7)	-0.3 (-2.4, 1.7)

Chemical	Unadjusted	Model 1: Adjusted for SES Variables[a]	Model 2: Adjusted for Perinatal, Maternal, and Caregiving Variables[b]	Model 3: Fully Adjust[c]	Model 4: Single Pollutant Models, No Restriction for Exposures
PCB 167 [d]	-2.6 (-5.3, 0.1)	1.2 (-1.0, 3.5)	-0.1 (-2.1, 2.0)	0.3 (-1.8, 2.5)	0.1 (-1.9, 2.2)
PCB 170	-5.3 (-8.5, -2.1)	-1.1 (-3.8, 1.6)	-1.4 (-4.7, 1.9)	-1.3 (-4.2, 1.7)	-1.2 (-4.2, 1.8)
PCB 172 [d]	-5.3 (-7.5, -3.0)	-1.9 (-4.1, 0.2)	-1.8 (-3.7, 0.1)	-1.3 (-3.4, 0.9)	-1.1 (-3.2, 0.9)
PCB 177 [d]	-3.3 (-6.0, -0.6)	-0.5 (-2.6, 1.7)	-0.7 (-2.7, 1.3)	-0.5 (-2.5, 1.4)	-0.6 (-2.5, 1.3)
PCB 178 [d]	-5.5 (-8.0, -3.0)	-1.8 (-4.0, 0.4)	-2.8 (-4.8, -0.8)	-2.6 (-4.7, -0.4)	-2.3 (-4.4, -0.2)
PCB 183	-3.1 (-5.3, -1.0)	0.0 (-2.2, 2.2)	-0.6 (-2.8, 1.7)	-0.2 (-2.3, 2.0)	-0.5 (-2.7, 1.7)
PCB 187	-4.1 (-6.5, -1.7)	-1.0 (-3.1, 1.2)	-1.4 (-3.8, 1.1)	-1.3 (-3.5, 0.9)	-1.2 (-3.4, 1.0)
PCB 194	-6.8 (-9.5, -4.1)	-2.2 (-4.7, 0.3)	-2.8 (-5.4, -0.2)	-2.5 (-4.9, 0.0)	-2.8 (-5.4, -0.2)
PCB 195 [d]	-3.8 (-6.2, -1.4)	-0.7 (-2.7, 1.4)	-0.5 (-2.5, 1.6)	-0.2 (-2.3, 1.9)	0.0 (-2.0, 2.1)
PCB 196/203	-4.3 (-7.0, -1.6)	-0.1 (-2.6, 2.5)	-0.3 (-3.1, 2.5)	0.0 (-2.5, 2.6)	0.0 (-2.5, 2.6)
PCB 199	-5.3 (-8.2, -2.5)	-0.8 (-3.7, 2.1)	-1.1 (-3.7, 1.6)	-1.1 (-3.7, 1.5)	-0.8 (-3.4, 1.8)
PCB 206	-3.0 (-6.1, 0.1)	-0.4 (-2.8, 2.0)	0.8 (-1.7, 3.3)	0.7 (-1.8, 3.1)	0.6 (-1.8, 3.0)
PCB 209	-4.3 (-6.6, -2.0)	-0.4 (-2.3, 1.6)	0.3 (-1.8, 2.4)	0.5 (-1.6, 2.7)	0.8 (-1.3, 2.9)
β-HCH [d]	-3.2 (-5.6, -0.8)	-1.2 (-3.4, 1.0)	-2.3 (-4.2, -0.4)	-2.3 (-4.4, -0.2)	-2.4 (-4.4, -0.3)
HCB	1.3 (-0.8, 3.3)	2.1 (0.0, 4.2)	2.0 (-0.3, 4.4)	2.2 (0.1, 4.4)	2.0 (0.0, 4.1)
p′p′-DDT [d]	2.2 (-0.4, 4.7)	1.7 (-0.4, 3.9)	1.3 (-0.7, 3.2)	1.1 (-0.8, 3.1)	1.1 (-0.8, 3.1)
p′p′-DDE	-1.4 (-3.5, 0.6)	0.5 (-1.6, 2.5)	-0.2 (-2.1, 1.8)	0.4 (-1.5, 2.3)	0.2 (-1.7, 2.1)
Oxychlordane	-1.2 (-3.4, 0.9)	2.1 (-0.1, 4.2)	1.7 (-0.7, 4.1)	2.3 (0.0, 4.6)	2.1 (0.0, 4.3)
trans-Nonachlor	-0.6 (-2.9, 1.8)	2.7 (0.4, 4.9)	2.2 (0.1, 4.3)	2.7 (0.5, 4.8)	2.5 (0.5, 4.6)
BB 153	-4.1 (-7.0, -1.2)	-0.4 (-2.5, 1.7)	-0.1 (-2.4, 2.3)	-0.1 (-2.2, 2.1)	0.4 (-1.8, 2.5)
PBDE 28	4.5 (1.9, 7.0)	2.9 (0.6, 5.3)	1.6 (-0.3, 3.5)	1.8 (-0.2, 3.7)	1.4 (-0.6, 3.3)
PBDE 47	5.5 (2.6, 8.4)	1.8 (-0.6, 4.2)	1.4 (-0.6, 3.4)	1.1 (-0.9, 3.1)	0.8 (-1.2, 2.7)
PBDE 85 [d]	2.1 (-0.4, 4.7)	-0.2 (-2.3, 1.9)	-0.5 (-2.5, 1.5)	-0.5 (-2.4, 1.4)	-0.7 (-2.6, 1.2)
PBDE 99	5.6 (2.7, 8.5)	1.4 (-1.1, 3.9)	1.1 (-1.1, 3.2)	0.7 (-1.3, 2.8)	0.7 (-1.4, 2.8)

Chemical	Unadjusted	Model 1: Adjusted for SES Variables[a]	Model 2: Adjusted for Perinatal, Maternal, and Caregiving Variables[b]	Model 3: Fully Adjust[c]	Model 4: Single Pollutant Models, No Restriction for Exposures
PBDE 100	4.0 (1.3, 6.7)	1.5 (-0.8, 3.8)	0.8 (-1.2, 2.7)	0.8 (-1.1, 2.7)	0.5 (-1.5, 2.4)
PBDE 153	0.8 (-1.5, 3.1)	1.2 (-0.8, 3.2)	0.3 (-1.6, 2.2)	0.6 (-1.2, 2.4)	0.4 (-1.4, 2.1)
PBDE 154 [d]	3.1 (0.4, 5.9)	0.8 (-1.4, 2.9)	0.6 (-1.5, 2.6)	0.4 (-1.6, 2.4)	0.2 (-1.9, 2.2)
PFOA	-0.8 (-3.0, 1.5)	0.5 (-1.6, 2.6)	-0.9 (-3.4, 1.5)	-0.9 (-3.1, 1.4)	-1.5 (-3.8, 0.7)
PFOS	0.1 (-2.3, 2.6)	2.2 (-0.1, 4.4)	1.9 (0.0, 3.8)	2.1 (0.2, 3.9)	1.1 (-1.5, 3.8)
PFNA	-0.1 (-2.6, 2.4)	2.6 (0.3, 4.9)	1.4 (-0.6, 3.4)	1.9 (-0.2, 4.0)	1.0 (-1.4, 3.5)
PFHxS	-1.2 (-3.8, 1.4)	2.3 (-0.2, 4.8)	0.4 (-1.5, 2.3)	1.0 (-1.1, 3.1)	1.0 (-1.3, 3.2)

[a]Adjusted for maternal race (white, black, and other), marital status (married, unmarried living with partner, and unmarried living alone), education (graduate/professional school/bachelor's degree, some college, high school, and < high school), insurance status during gestation (private and public/no insurance), employment during pregnancy (any and non-employed), and household income (continuous in $10,000 increments). [b]Adjusted for maternal age (continuous, years), parity (0, 1-2, and 3+), prenatal vitamin use (daily, 1-6 times/week, and never or rarely), maternal IQ (continuous), depressive symptoms during pregnancy (continuous), HOME score (continuous), and gestational serum cotinine concentration (continuous $\log_{10}$ transformed). [c]Adjusted for both sets of covariates in c and d. [d]The displayed betas are the change in SRS scores among children born to women with detectable vs. non-detectable levels of these chemicals. All other chemicals were treated as continuous $\log_{10}$-transformed variables that are divided by two times their standard deviation to put them on a comparable scale to the dichotomous variables.

Abbreviations: MBP-Mono-n-butyl phthalate, MiBP-Mono-iso-butyl phthalate, MEP-Monoethyl phthalate, MBzP-Monoebenzyl phthalate, MCPP-Mono-3-carboxypropyl phthalate, MEHP-Mono-2-ethylhexyl phthalate, MEHHP, Mono-2-ethyl-5-hydroxyhexyl phthalate, MECPP-mono-2-ethyl-5-carboxypentyl phthalate, BPA-Bisphenol A, PCB-Polychlorinated biphenyl, HCH-Hexachlorocyclohexane, HCB-Hexachlorobenzene, DDT -dichlorodiphenyltrichloroethane, DDE- dichlorodiphenyldichloroethylene, BB-Brominated biphenyl, PBDE-Polybrominated diphenyl ether, PFOA-Perfluoroctanoate, PFOS-Perfluorooctane sulfonate, PFNA-Perfluorononanoate, PFHxS-Perfluorohexane sulfonate

Table S5. Covariate adjusted, multi-pollutant models [β (95% CI)] of endocrine disrupting chemicals and SRS Total T scores: Assessing model sensitivity.[a]

Chemical	Multi-Pollutant Hierarchical Model: $t^2 = 26$[b]	Multi-Pollutant Hierarchical Model: $t^2 = 6.5$[c] (Results in Figure 2)	Multi-Pollutant Hierarchical Model: Empirical Bayes[d]	Multi-Pollutant Model: Restricted Set of Chemicals[e]	Multi-Pollutant Hierarchical Model: IPWs for Loss to Follow-Up
MBP	-1.2 (-3.4, 0.9)	-1.4 (-3.3, 0.4)	-1.3 (-3.0, 0.4)	-1.1 (-3.3, 1.2)	-1.3 (-3.4, 0.8)
MiBP	0.7 (-1.4, 2.8)	0.8 (-1.1, 2.7)	0.7 (-0.9, 2.4)	0.8 (-1.2, 2.9)	0.8 (-1.2, 2.8)
MEP	-0.9 (-2.6, 0.7)	-0.7 (-2.3, 0.8)	-0.5 (-2.0, 0.9)	-1.1 (-2.7, 0.6)	-1.0 (-2.6, 0.6)
MBzP	-0.8 (-2.9, 1.3)	-0.5 (-2.5, 1.4)	-0.3 (-2.0, 1.4)	-1.0 (-3.2, 1.2)	-0.7 (-2.8, 1.4)
MCPP	0.2 (-1.6, 2.1)	0.4 (-1.3, 2.0)	0.4 (-1.2, 1.9)	0.4 (-1.4, 2.3)	0.2 (-1.6, 2.1)
MEHP	0.7 (-2.5, 3.9)	0.4 (-2.2, 2.9)	0.5 (-1.7, 2.6)	-0.3 (-3.2, 2.6)	0.6 (-2.6, 3.8)
MECPP	1.6 (-2.6, 5.8)	1.1 (-2.0, 4.1)	0.8 (-1.6, 3.3)	1.0 (-1.9, 3.8)	1.6 (-2.5, 5.8)
MEHHP	-1.7 (-6.3, 2.9)	-0.5 (-3.6, 2.7)	-0.1 (-2.7, 2.5)	Not Included	-1.7 (-6.2, 2.8)
BPA	0.5 (-1.6, 2.6)	0.6 (-1.2, 2.5)	0.7 (-1.0, 2.4)	0.6 (-1.5, 2.8)	0.7 (-1.3, 2.8)
PCB 28	0.0 (-2.4, 2.5)	-0.4 (-2.4, 1.7)	-0.3 (-2.0, 1.4)	-0.4 (-2.9, 2.0)	-0.1 (-2.5, 2.3)
PCB 66 [f]	0.7 (-2.0, 3.4)	0.6 (-1.8, 3.0)	0.4 (-1.6, 2.4)	1.0 (-1.8, 3.8)	0.7 (-2.0, 3.4)
PCB 74	-3.9 (-8.0, 0.1)	-2.2 (-5.2, 0.9)	-1.2 (-3.8, 1.3)	-3.2 (-7.2, 0.9)	-3.8 (-7.8, 0.2)
PCB 99	-1.9 (-6.9, 3.0)	-0.8 (-4.1, 2.6)	-0.3 (-3.0, 2.5)	0.9 (-3.2, 5.0)	-2.0 (-6.9, 2.9)
PCB 101 [f]	-0.1 (-2.4, 2.1)	-0.3 (-2.1, 1.6)	-0.3 (-1.9, 1.3)	-0.1 (-2.3, 2.1)	-0.2 (-2.4, 2.0)
PCB 105	0.7 (-2.3, 3.7)	0.6 (-1.9, 3.0)	0.5 (-1.6, 2.5)	0.7 (-2.4, 3.7)	0.7 (-2.2, 3.6)
PCB 118	-1.4 (-2.9, 0.1)	-1.0 (-2.4, 0.3)	-0.8 (-2.0, 0.4)	-1.0 (-2.6, 0.5)	-1.5 (-3.0, 0.0)
PCB 138/158	6.6 (1.1, 12.2)	3.4 (-0.2, 7.0)	2.3 (-1.0, 5.5)	Not Included	6.6 (1.1, 12.1)
PCB 146	0.1 (-3.3, 3.4)	-0.1 (-2.8, 2.6)	-0.1 (-2.3, 2.0)	-0.2 (-3.6, 3.2)	0.1 (-3.2, 3.4)
PCB 153	-5.1 (-10.6, 0.4)	-2.6 (-6.3, 1.1)	-1.6 (-4.8, 1.5)	-1.9 (-7.0, 3.3)	-5.0 (-10.4, 0.5)
PCB 156	1.1 (-2.9, 5.2)	0.4 (-2.7, 3.5)	0.1 (-2.4, 2.6)	1.5 (-2.5, 5.5)	1.0 (-3.0, 5.0)
PCB 157 [f]	-0.8 (-3.5, 1.9)	-0.2 (-2.5, 2.1)	0.0 (-2.0, 1.9)	-0.6 (-3.4, 2.2)	-1.0 (-3.6, 1.7)

Chemical	Multi-Pollutant Hierarchical Model: $t^2 = 26$[b]	Multi-Pollutant Hierarchical Model: $t^2 = 6.5$[c] (Results in Figure 2)	Multi-Pollutant Hierarchical Model: Empirical Bayes[d]	Multi-Pollutant Model: Restricted Set of Chemicals[e]	Multi-Pollutant Hierarchical Model: IPWs for Loss to Follow-Up
PCB 167 [f]	1.3 (-1.3, 3.9)	1.0 (-1.3, 3.2)	0.6 (-1.3, 2.6)	1.2 (-1.4, 3.9)	1.4 (-1.2, 3.9)
PCB 170	1.5 (-4.1, 7.0)	1.0 (-2.7, 4.6)	0.5 (-2.5, 3.5)	Not Included	1.6 (-3.9, 7.0)
PCB 172 [f]	-0.7 (-3.9, 2.5)	-0.6 (-3.2, 2.0)	-0.6 (-2.7, 1.5)	-0.5 (-3.7, 2.6)	-0.5 (-3.7, 2.6)
PCB 177 [f]	0.9 (-2.0, 3.8)	0.4 (-1.9, 2.8)	0.1 (-1.9, 2.1)	0.6 (-2.3, 3.5)	0.9 (-1.9, 3.7)
PCB 178 [f]	-3.0 (-6.3, 0.2)	-2.7 (-5.3, -0.1)	-2.2 (-4.3, -0.1)	-3.0 (-6.4, 0.3)	-2.9 (-6.1, 0.2)
PCB 183	0.9 (-2.1, 3.8)	0.2 (-2.2, 2.6)	0.0 (-2.1, 2.1)	0.6 (-2.4, 3.6)	0.8 (-2.1, 3.7)
PCB 187	-1.1 (-4.6, 2.4)	-0.7 (-3.4, 2.1)	-0.6 (-2.9, 1.6)	-0.9 (-4.4, 2.6)	-1.4 (-4.8, 2.1)
PCB 194	-3.4 (-7.5, 0.7)	-2.6 (-5.7, 0.4)	-2.0 (-4.5, 0.5)	-3.0 (-7.0, 1.0)	-3.8 (-7.9, 0.2)
PCB 195 [f]	0.2 (-2.8, 3.2)	0.4 (-2.1, 2.9)	0.4 (-1.6, 2.5)	0.6 (-2.4, 3.6)	-0.1 (-3.0, 2.9)
PCB 196/203	2.2 (-1.9, 6.4)	1.3 (-1.9, 4.4)	0.8 (-1.8, 3.3)	2.3 (-1.7, 6.2)	2.4 (-1.6, 6.5)
PCB 199	-1.6 (-6.8, 3.7)	-1.1 (-4.7, 2.5)	-0.7 (-3.6, 2.1)	Not Included	-1.1 (-6.4, 4.2)
PCB 206	1.0 (-1.6, 3.7)	1.3 (-0.9, 3.6)	1.1 (-0.8, 3.0)	0.0 (-3.0, 2.9)	1.1 (-1.6, 3.8)
PCB 209 [f]	1.6 (-1.5, 4.6)	0.8 (-1.7, 3.2)	0.5 (-1.6, 2.6)	1.2 (-1.7, 4.1)	1.4 (-1.6, 4.4)
β-HCH [f]	-3.3 (-6.1, -0.5)	-3.2 (-5.5, -0.9)	-2.6 (-4.5, -0.6)	-3.7 (-6.5, -0.9)	-3.2 (-6.0, -0.4)
HCB	1.8 (-0.9, 4.4)	1.6 (-0.6, 3.8)	1.4 (-0.5, 3.2)	1.8 (-0.9, 4.5)	1.9 (-0.7, 4.5)
p′p′-DDT [f]	1.0 (-1.8, 3.8)	0.9 (-1.4, 3.2)	0.8 (-1.2, 2.7)	1.0 (-1.9, 3.9)	1.2 (-1.7, 4.0)
p′p′-DDE	1.5 (-1.1, 4.1)	1.1 (-1.1, 3.2)	0.8 (-1.1, 2.7)	1.6 (-0.8, 4.1)	1.6 (-1.0, 4.2)
Oxychlordane	-1.6 (-4.7, 1.5)	-0.9 (-3.4, 1.6)	-0.3 (-2.3, 1.8)	-1.2 (-4.3, 2.0)	-1.6 (-4.7, 1.5)
trans-Nonachlor	4.1 (0.8, 7.3)	3.3 (0.8, 5.9)	2.5 (0.3, 4.7)	3.8 (0.8, 6.8)	3.9 (0.7, 7.2)
BB 153	-1.1 (-3.3, 1.1)	-1.1 (-3.2, 1.0)	-1.0 (-3.0, 0.9)	-1.2 (-3.6, 1.1)	-1.0 (-3.2, 1.2)
PBDE 28	2.5 (-0.6, 5.6)	2.2 (-0.3, 4.7)	1.7 (-0.4, 3.7)	2.6 (-0.5, 5.8)	2.3 (-0.8, 5.4)
PBDE 47	1.0 (-4.7, 6.6)	0.5 (-3.1, 4.1)	0.4 (-2.6, 3.3)	1.8 (-2.9, 6.4)	0.6 (-5.0, 6.2)
PBDE 85 [f]	-3.2 (-5.9, -0.5)	-2.4 (-4.6, -0.1)	-1.8 (-3.8, 0.1)	-2.3 (-5.0, 0.5)	-3.1 (-5.8, -0.4)

Chemical	Multi-Pollutant Hierarchical Model: $t^2 = 26$[b]	Multi-Pollutant Hierarchical Model: $t^2 = 6.5$[c] (Results in Figure 2)	Multi-Pollutant Hierarchical Model: Empirical Bayes[d]	Multi-Pollutant Model: Restricted Set of Chemicals[e]	Multi-Pollutant Hierarchical Model: IPWs for Loss to Follow-Up
PBDE 99	1.7 (-2.5, 5.9)	0.6 (-2.4, 3.7)	0.3 (-2.2, 2.8)	Not Included	1.8 (-2.3, 5.9)
PBDE 100	-2.8 (-8.0, 2.3)	-1.5 (-5.0, 2.1)	-0.9 (-3.8, 2.0)	-2.8 (-8.0, 2.4)	-2.5 (-7.5, 2.5)
PBDE 153	0.9 (-1.9, 3.8)	0.2 (-2.1, 2.4)	0.0 (-1.9, 1.9)	0.3 (-2.6, 3.3)	0.8 (-2.0, 3.6)
PBDE 154 [f]	-0.4 (-3.3, 2.6)	-0.1 (-2.5, 2.3)	0.1 (-1.9, 2.1)	-0.2 (-3.2, 2.8)	-0.4 (-3.3, 2.5)
PFOA	-2.0 (-4.4, 0.4)	-1.6 (-3.7, 0.5)	-1.4 (-3.2, 0.5)	-2.1 (-4.5, 0.4)	-1.9 (-4.3, 0.5)
PFOS	1.6 (-0.8, 4.1)	1.1 (-1.0, 3.2)	0.8 (-1.1, 2.6)	1.6 (-1.0, 4.1)	1.8 (-0.7, 4.2)
PFNA	-0.1 (-2.8, 2.6)	0.0 (-2.2, 2.2)	0.0 (-1.9, 1.9)	0.4 (-2.4, 3.1)	-0.3 (-2.9, 2.4)
PFHxS	1.0 (-1.2, 3.3)	0.1 (-1.8, 2.1)	-0.3 (-2.0, 1.5)	1.3 (-1.2, 3.7)	1.0 (-1.3, 3.2)

[a]All models adjusted for maternal age (continuous in years), race (white, black, and other), marital status (married, unmarried living with partner, and unmarried living alone), education (graduate/professional school/bachelor's degree, some college, high school, and < high school), parity (0, 1-2, and 3+), insurance status during gestation (private and public/no insurance), prenatal vitamin use (daily, 1-6 times/week, and never or rarely), employment during pregnancy (any and non-employed), maternal IQ (continuous), household income (continuous in \$10,000 increments), depressive symptoms during pregnancy (continuous), HOME score (continuous), and gestational serum cotinine concentration (continuous $\log_{10}$ transformed). [b]The residual variance (τ^2) of the semi-Bayes model was set to 26.03, corresponds to 95% confidence that the true effect of a chemical on SRS scores lies between -10 to 10. The exchangeability matrix is contains an intercept and indicator (0/1) variables for OCs, PCBs, BFRs, PFASs, DEHP metabolites, DBP metabolites, and persistent vs. non-persistent chemicals. [c]The residual variance (τ^2) of the semi-Bayes model was set to 6.5, corresponds to 95% confidence that the true effect of a chemical on SRS scores lies between -5 to 5. The exchangeability matrix is contains an intercept and indicator (0/1) variables for OCs, PCBs, BFRs, PFASs, DEHP metabolites, DBP metabolites, and persistent vs. non-persistent chemicals. [d]The residual variance is estimated from the data (i.e., $\tau^2 = 0$). [e]Restricted to chemicals whose concentrations with all other chemicals was ≤ 0.90 (Pearson R). [f]The displayed betas are the change in SRS scores among children born to women with detectable vs. non-detectable levels of these chemicals.

All other chemicals were treated as continuous $\log_{10}$-transformed variables that are divided by two times their standard deviation to put them on a comparable scale to the dichotomous variables.

Abbreviations-MBP-Mono-n-butyl phthalate, MiBP-Mono-iso-butyl phthalate, MEP-Monoethyl phthalate, MBzP-Monoebenzyl phthalate, MCPP-Mono-3-carboxypropyl phthalate, MEHP-Mono-2-ethylhexyl phthalate, MEHHP, Mono-2-ethyl-5-hydroxyhexyl phthalate, MECPP-mono-2-ethyl-5-carboxypentyl phthalate, BPA-Bisphenol A, PCB-Polychlorinated biphenyl, HCH-Hexachlorocyclohexane, HCB-Hexachlorobenzene, DDT-dichlorodiphenyltrichloroethane, DDE- dichlorodiphenyldichloroethylene, BB-Brominated biphenyl, PBDE-Polybrominated diphenyl ether, PFOA-Perfluoroctanoate, PFOS-Perfluorooctane sulfonate, PFNA-Perfluorononanoate, PFHxS-Perfluorohexane sulfonate, SB-Semi-Bayes, IPW-Inverse Probability Weights

Table S6. Adjusted sex-specific change in SRS Total T scores in 4 and 5 year old Cincinnati children with increasing maternal gestational urinary or serum endocrine disrupting chemical concentrations (n = 175).[a]

Chemical	Girls: b (95% CI)	Boys: b (95% CI)	Interaction p-value
MBP	-0.6 (-3.2, 2.0)	-0.2 (-2.6, 2.3)	0.83
MiBP	-0.6 (-3.5, 2.4)	1.1 (-1.1, 3.2)	0.39
MEP	1.1 (-1.7, 3.9)	-1.9 (-3.9, 0.1)	0.09
MBzP	-0.5 (-4.0, 2.9)	-0.7 (-3.0, 1.6)	0.93
MCPP	0.9 (-2.0, 3.9)	1.2 (-1.1, 3.6)	0.87
MEHP	1.5 (-1.0, 3.9)	1.3 (-1.2, 3.8)	0.93
MECPP	2.3 (-0.5, 5.0)	0.9 (-1.3, 3.1)	0.45
MEHHP	1.2 (-1.6, 4.1)	1.0 (-1.3, 3.4)	0.93
BPA	2.6 (-0.9, 6.1)	0.0 (-2.3, 2.3)	0.22
PCB 28	0.3 (-2.3, 3.0)	1.9 (-0.5, 4.3)	0.40
PCB 66[c]	1.3 (-1.9, 4.4)	0.5 (-2.7, 3.8)	0.73
PCB 74	-0.6 (-3.4, 2.2)	-0.5 (-3.1, 2.1)	0.94
PCB 99	-0.1 (-2.9, 2.7)	0.2 (-2.4, 2.7)	0.90
PCB 101[c]	-0.1 (-3.1, 3.0)	-0.3 (-2.8, 2.3)	0.88
PCB 105	0.9 (-2.8, 4.6)	0.0 (-2.1, 2.2)	0.66
PCB 118	-0.6 (-4.7, 3.5)	-0.3 (-2.0, 1.4)	0.92
PCB 138/158	0.0 (-2.6, 2.6)	-0.6 (-3.6, 2.5)	0.75
PCB 146	-1.2 (-4.7, 2.2)	-0.5 (-3.5, 2.4)	0.72
PCB 153	-1.5 (-4.8, 1.8)	-1.4 (-4.3, 1.6)	0.92
PCB 156	-1.7 (-5.3, 1.9)	-0.5 (-3.7, 2.7)	0.56
PCB 157[c]	-0.6 (-3.7, 2.4)	-0.3 (-2.9, 2.3)	0.86
PCB 167[c]	0.5 (-2.4, 3.4)	0.1 (-2.5, 2.7)	0.82
PCB 170	-0.9 (-4.4, 2.5)	-2.1 (-5.3, 1.1)	0.58
PCB 172[c]	-1.3 (-4.4, 1.9)	-1.3 (-3.6, 1.1)	0.98
PCB 177[c]	-1.2 (-3.9, 1.6)	0.2 (-2.4, 2.7)	0.46
PCB 178[c]	-3.4 (-6.2, -0.6)	-1.7 (-4.3, 0.9)	0.32
PCB 183	-0.4 (-3.2, 2.5)	0.2 (-2.6, 2.9)	0.77
PCB 187	-1.0 (-3.8, 1.9)	-1.9 (-4.2, 0.4)	0.60
PCB 194	-1.6 (-5.0, 1.7)	-3.5 (-5.9, -1.0)	0.33
PCB 195 [c]	0.5 (-2.4, 3.4)	-0.9 (-3.4, 1.6)	0.45

Chemical	Girls: b (95% CI)	Boys: b (95% CI)	Interaction p-value
PCB 196/203	0.8 (-2.6, 4.3)	-1.1 (-3.7, 1.6)	0.36
PCB 199	-1.0 (-4.5, 2.4)	-1.1 (-4.1, 1.9)	0.99
PCB 206	-0.3 (-3.8, 3.1)	1.8 (-1.1, 4.8)	0.32
PCB 209[c]	0.4 (-2.7, 3.4)	0.7 (-2.0, 3.4)	0.84
β-HCH [c]	-2.3 (-5.4, 0.9)	-2.4 (-4.9, 0.1)	0.96
HCB	4.9 (1.9, 7.8)	0.3 (-1.9, 2.6)	0.02
p′p′-DDT [c]	2.3 (-0.5, 5.2)	-0.2 (-2.8, 2.3)	0.18
p′p′-DDE	0.3 (-2.6, 3.3)	0.5 (-1.7, 2.7)	0.93
Oxychlordane	3.6 (0.3, 7.0)	1.3 (-1.2, 3.9)	0.25
trans-Nonachlor	5.6 (2.4, 8.8)	0.3 (-1.7, 2.2)	<0.01
BB 153	-0.6 (-3.9, 2.6)	0.3 (-2.3, 3.0)	0.64
PBDE 28	3.0 (0.2, 5.8)	0.4 (-2.3, 3.1)	0.18
PBDE 47	1.7 (-1.1, 4.5)	0.5 (-2.5, 3.5)	0.56
PBDE 85 [c]	0.5 (-2.3, 3.3)	-1.6 (-4.1, 0.9)	0.29
PBDE 99	0.5 (-2.3, 3.3)	-1.6 (-4.1, 0.9)	0.60
PBDE 100	1.6 (-1.4, 4.5)	0.2 (-2.5, 2.8)	0.48
PBDE 153	1.3 (-1.5, 4.2)	-0.2 (-2.6, 2.2)	0.43
PBDE 154[c]	1.1 (-1.9, 4.1)	-0.4 (-3.1, 2.3)	0.48
PFOA	-1.8 (-4.6, 1.0)	0.7 (-2.5, 3.8)	0.22
PFOS	0.9 (-1.5, 3.3)	3.8 (1.3, 6.3)	0.08
PFNA	1.4 (-1.6, 4.4)	2.6 (0.0, 5.2)	0.54
PFHxS	1.6 (-1.1, 4.2)	0.4 (-2.4, 3.2)	0.51

[a]Adjusted for maternal age (continuous in years), race (white, black, and other), marital status (married, unmarried living with partner, and unmarried living alone), education (graduate/professional school/bachelor's degree, some college, high school, and < high school), parity (0, 1-2, and 3+), insurance status during gestation (private and public/no insurance), prenatal vitamin use (daily, 1-6 times/week, and never or rarely), employment during pregnancy (any and non-employed), IQ (continuous), household income (continuous in $10,000 increments), depressive symptoms during pregnancy (continuous), HOME score (continuous), and gestational serum cotinine concentration (continuous, $\log_{10}$ transformed). [b]d-CLD is the confidence limit difference, which is the upper 95% CL minus the lower 95% CL. [c]The displayed betas are the change in SRS scores among children born to women with detectable vs.

non-detectable levels of these chemicals. All other chemicals were treated as continuous log_{10}-transformed variables that are divided by two times their standard deviation to put them on a comparable scale to the dichotomous variables.

Abbreviations: MBP-Mono-n-butyl phthalate, MiBP-Mono-iso-butyl phthalate, MEP-Monoethyl phthalate, MBzP-Monoebenzyl phthalate, MCPP-Mono-3-carboxypropyl phthalate, MEHP-Mono-2-ethylhexyl phthalate, MEHHP, Mono-2-ethyl-5-hydroxyhexyl phthalate, MECPP-mono-2-ethyl-5-carboxypentyl phthalate, BPA-Bisphenol A, PCB-Polychlorinated biphenyl, HCH-Hexachlorocyclohexane, HCB-Hexachlorobenzene, -dichlorodiphenyltrichloroethane, DDE- dichlorodiphenyldichloroethylene, BB-Brominated biphenyl, PBDE-Polybrominated diphenyl ether, PFOA-Perfluorooctanoate, PFOS-Perfluorooctane sulfonate, PFNA-Perfluorononanoate, and PFHxS-Perfluorohexane sulfonate.
